# Synthesis of novel hexamolybdenum cluster-functionalized copper hydroxide nanocomposites and its catalytic activity for organic molecule degradation

**DOI:** 10.1080/14686996.2021.1961559

**Published:** 2021-09-15

**Authors:** Thi Kim Ngan Nguyen, Cédric Bourgès, Takashi Naka, Fabien Grasset, Noée Dumait, Stéphane Cordier, Takao Mori, Naoki Ohashi, Tetsuo Uchikoshi

**Affiliations:** a Research Center for Functional Materials, National Institute for Materials Science (NIMS), Ibaraki, Japan; b Laboratory for Innovative Key Materials and Structures (LINK), National Institute for Materials Science (NIMS), Ibaraki, Japan; c WPI International Center for Materials Nanoarchitechtonics (WPI-MANA), National Institute for Materials Science (NIMS), Tsukuba, Japan; d University Rennes-CNRS, UMR6226, Institut des Sciences Chimiques de Rennes (ISCR), Rennes, France

**Keywords:** Molybdenum octahedral clusters, copper hydroxide nitrate, oxidation catalyst, hydroxide peroxide, dye degradation, 10 Engineering and Structural materials, 102 Porous / Nanoporous / Nanostructured materials, 103 Composites, 105 Low-Dimension (1D/2D) materials, 205 Catalyst / Photocatalyst / Photosynthesis, 301 Chemical syntheses / processing, 501 Chemical analyses, 204 Optics / Optical applications

## Abstract

A novel heterogeneous catalytic nanomaterial based on a molybdenum cluster-based halide (MC) and a single-layered copper hydroxynitrate (CHN) was first prepared by colloidal processing under ambient conditions. The results of the elemental composition and crystalline pattern indicated that CHN was comprehensively synthesized with the support of the MC compound. The absorbing characteristic in the ultraviolet and near-infrared regions was promoted by both of the ingredients. The proper chemical interaction between the materials is a crucial reason to modify the structure of the MCs and only a small decrease in the magnetic susceptibility of CHN. The heterogeneous catalytic activity of the obtained MC@CHN material was found to have a high efficiency and excellent reuse when it is activated by hydrogen peroxide (H_2_O_2_) for the degrading reaction of the organic pollutant at room temperature. A reasonable catalytic mechanism was proposed to explain the distinct role of the copper compound, Mo_6_ compound, and H_2_O_2_ in the production of the radical hydroxyl ion. This novel nanomaterial will be an environmentally promising candidate for dye removal.

## Graphical abstract

**Figure:**
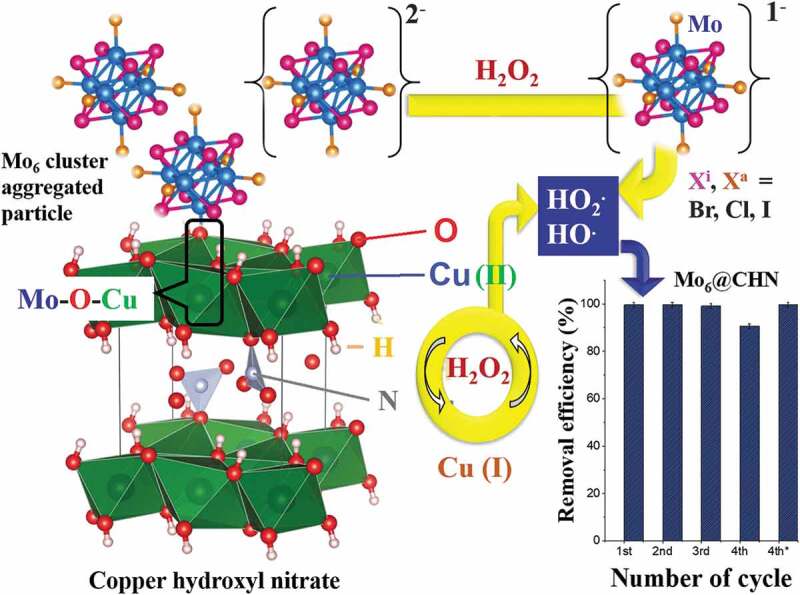

## 1. Introduction

The controlled assembling of several inorganic phases enables the creation of nanocomposites with versatile physical and chemical properties for possible integration in various multi-property devices [1,2]. Material engineering has developed a set of methodologies to design the various families of functional materials by applying solid-state chemistry, molecular chemistry, colloidal processing, or biochemistry. The octahedral molybdenum cluster halides with the general formula of A_2_[{Mo_6_X^i^_8_}X^a^_6_] (A = Cs, K, or alkylammonium cations; X = Cl, Br, or I) constitute a representative family of polyversatile integrable compounds into inorganic nanocomposites [3]. A_2_[{Mo_6_X^i^_8_}X^a^_6_] is based on the unique structured [{Mo_6_X^i^_8_}X^a^_6_]^2-^ cluster unit that is used as functional building blocks that are synthesized by solid-state chemistry and wet solution chemistry [4]. The latter contains the Mo_6_ clusters with the Mo-Mo bonds that are stabilized by face-capping ligands (X^i^) and terminal ligands (X^a^). From an electronic point of view, the [{Mo_6_X^i^_8_}X^a^_6_]^2-^ cluster unit exhibits a closed 24 valence electron shell. The understanding of the intrinsic natures of redox transitions, photochemistry, or electrochemistry has motivated the research by several groups around the world [5–9]. The discovery of the unique photoactive and oxidative characteristics has broadened the potential applications to the fields of catalysis [10,11], optoelectronics [12,13], and biology [14–16]. The photoluminescence of the [{Mo_6_X^i^_8_}X^a^_6_]^2-^ cluster unit in a large red-NIR window originates from the important geometrical relaxations occurring in the triplet states. These geometric relaxations follow two possible deformations when the octahedral clusters are photo-excited. These deformations correspond to either the elongation of the Mo_6_ octahedron along the pseudo-4 fold axis or to the elongation of a Mo – Mo bond. One or the other of these deformations are preferred depending on the environment of the [{Mo_6_X^i^_8_}X^a^_6_]^2-^ blocks (counterions, crystal packing) [9]. As a phosphorescent dye, another characteristic of the [{Mo_6_X^i^_8_}X^a^_6_]^2-^ cluster units is their capability to produce singlet oxygen (^1^O_2_) [8,17]. The catalytic characteristic of the halide [{Mo_6_X^i^_8_}X^a^_6_]^2-^ cluster units was approached following two pathways, i.e. i) a photoactive catalyst based on the photoexcited cluster generating a pair of holes and electrons [10,18] and ii) redox catalyst based on the oxidized [{Mo_6_X_8_}X_6_]^1−^state as a strong and powerful oxidant [6,11]. For instance, the mixed halide [{Mo_6_I_8_}Cl_6_]^2-^ cluster unit can be reversibly oxidized [6]. Based on this behavior, the recycling ability of the heterogeneous catalyst based on the Mo_6_ cluster has been developed. The physicochemical interactions between the metal clusters and inorganic matrix in the multi-component material are essential to control the structural characteristics and the catalytic mechanisms. The catalytic properties of the Mo-based MC have been developed in recent and relevant studies by utilizing the Ouzo effect originating from nanomarbles for increasing the HER activity [19] or incorporated with graphene for photocatalytic water reduction [20] and hydrogen evolution [21] with a high efficiency. The prominent photoactive and oxidation performance of the Mo_6_ cluster anchored on a layered double hydroxide activated by the hydrogen peroxide oxidizer has been revealed with a proper catalytic efficiency [11]. However, the reuse of the catalyst has not met the requirement.

For the first time, we now propose a new matrix based on the single-layered hydroxide salt (LHS) to promote the redox property of the Mo_6_ cluster-based halide. The obtained nanocomposites were synthesized by using a colloidal process under ambient conditions. The general formula of LHS is M(OH)_2−x_(A^n^^−^)_x/n_.mH_2_O, where M^2+^ is the metallic cation (e.g. Cu^2+^, Mg^2+^, Ni^2+^, Zn^2+^, and Ca^2+^) and A is a counterion (NO_3_^−^, SO_4_^2-^, Cl^−^ and acetate) with a negative charge n^−^ [22]. The advantageous LHS makes this compound useful as a strategic precursor for developing copper oxide nanostructures [23,24]. Indeed, a few LHS have been reported as promising catalysts for dye removal by an advanced oxidation process using the H_2_O_2_ oxidizer to produce the radical species; Zn_5_[OH]_8_(counterion)_2_/H_2_O_2_ or Cu_2_[OH]_3_(counterion)/H_2_O_2_ systems resulting in a high catalytic efficiency for organic dye bleaching [25,26] or layered copper hydroxynitrate (CHN) and CHN nanosheets/H_2_O_2_ for the recyclable enzyme-mimicking colorimetric sensor of biothiols [27]. In addition, the incorporations of LHS and supporting material systems have been studied for different application purposes such as CHN/ZnO [28] and anionic iron(III) porphyrins/Zn_5_[OH]_8_(counterion)_2_ [29] for dye degradation, CHN/Cu_2_O for photocatalysis [30], or CHN for conductive copper thin-films [31]. Besides the huge potential for designing a heterogeneous catalyst due to its low toxicity, high catalytic characteristic, and reusability, the copper compound has attracted attention as a promising UV-NIR adsorbing pigment for smart window applications [32].

To the best of our knowledge, only the efficient photo-redox heterogeneous hybrid composite catalyst constituting the [{Re_6_S^i^_8_}(CN)^a^_6_]^4-^ cluster compounds and copper oxide-modified TiO_2_ have already been reported. It has been demonstrated by Kumar et al. that such an association leads to a good nanocomposite material for the reduction of CO_2_ under visible light irradiation [33]. The important point was noted that the hexanuclear rhenium clusters act as a sensitizer to the copper-modified TiO_2_ during the catalytic reactions.

Besides the improved catalytic activity, the reuse of catalysts also plays an important role in industrial applications. The incorporation of the different redox cluster units with a 1D, 2D or 3D structured material has progressed not only to understand the tunability of their redox and catalytic properties followed by the compositions and structural arrangements, but also to enhance their reuse. This study aimed to assemble a copper hydroxynitrate (CHN) and a cluster compound (MC) (i.e. A_2_[{Mo_6_X^i^_8_}X^a^_6_] (A = Cs, or alkylammonium cations; X = Cl, Br, or I) in the nanocomposite to improve the optical and catalytic properties for the heterogeneous catalysts in water treatment. Interestingly, the A_2_[{Mo_6_X^i^_8_}X^a^_6_] halide acted as a catalytic agent for the formation of the crystalline copper hydroxynitrate when wet solution chemistry has been coupled with heat treatment. The elemental composition, morphology, and optical and magnetic properties of the obtained materials were comprehensively investigated. Moreover, the proper catalytic property activated by the hydrogen peroxide (H_2_O_2_) and their reuse in the dye removal reaction of the obtained nanomaterial has been investigated.

## 2. Materials and methods

### 2.1. Materials

The Cu(NO_3_)_2_∙3H_2_O (99%), acetone (99.5%), and ethanol (99.5%) were commercially purchased from Nacalai Tesque. The ammonia solution (NH_3_, 28%) and hydrogen peroxide (30% in water) were supplied from Wako Pure Chemical Industries, Ltd. All chemicals were used without purification. The deionized water with the conductance of 0.5.10^−4^ S/m was obtained using Water Purifiers WG710 equipment at 25°C.

### 2.2. Preparation of metal cluster@copper hydroxynitrate

#### Synthesis of octahedral molybdenum cluster halides (MC)

The ((n-C_4_H_9_)_4_N)_2_[{Mo_6_Cl^i^_8_}Cl^a^_6_] **(1)** complex was synthesized by the wet chemistry reaction using Cs_2_[{Mo_6_Cl^i^_8_}Cl^a^_6_] **(2)** and ((n-C_4_H_9_)_4_N)Cl compounds according to previously published procedures [34,35]. The Cs_2_[{Mo_6_Cl^i^_8_}Cl^a^_6_] **(2)** was prepared by cationic metathesis in solution starting from CsCl and (H_3_O)_2_Mo_6_Cl_14_ [36]. The Cs_2_[{Mo_6_Br^i^_8_}Br^a^_6_] **(3)** and Cs_2_[{Mo_6_I^i^_8_}CI^a^_6_] **(4)** clusters were prepared by the reaction of CsX (X = Br, I) (Alfa Aesar 99.9%) and MoX_2_ (X = Br, I) at high temperature [36]. All the clusters were reproduced by permission from references [34-36], copyright [2005, Kirakci].

#### Preparation of the MC-functionalized copper hydroxynitrate nanocomposites

**Figure 1.**
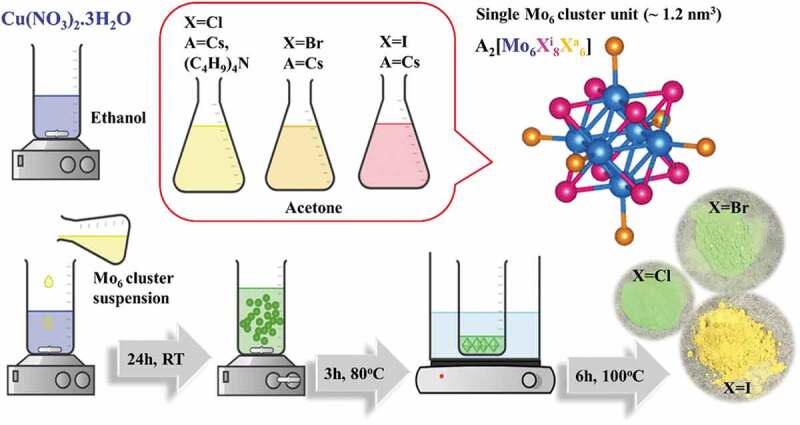
The schematic illustration of the [{Mo_6_X^i^_8_}X^a^_6_]^2-^ cluster unit (MC) and the preparation of the MC@CHN nanocomposites

Copper hydroxynitrate, abbreviated as CHN, was fabricated in the presence of the A_2_[{Mo_6_X^i^_8_}X^a^_6_] compounds (MCs) (A = Cs, or alkylammonium cations; X = Cl, Br or I) by the colloidal process under ambient conditions. Cu(NO_3_)_2_∙3H_2_O dissolved in ethanol (17 g/L) and the solution of MC in acetone (0.7 g/L) were separately obtained by using a magnetic stirrer with sonication for 1 h at room temperature. The MC solution was then slowly poured into the copper aqueous solution and stirred for 24 h to obtain a slight cloudy suspension. The four weight ratios of the different MCs and Cu(NO_3_)_2_∙3H_2_O in the aqueous solutions were 1:5, 1:10, 1:15 and 1:20, respectively, denoted as the 15, 110, 115 and 120 numbers after CHN (Table 1). Next, these solutions were put in a hot water bath, followed by thermal treatment to remove the solvent at 80°C until a dried green crystalline powder was obtained (Figure 1). Generally, the advantages of the present procedure include two steps: i) a simple mechanical mixture of the cluster and Cu(NO_3_)_2_.3H_2_O to first form tiny CHN crystals at RT, and ii) growth of the CHN crystal and performance of the nanocomposite with the cluster during stirring at 80°C in air. The MC@CHN products were completely dried at 100°C for 6 h, then washed several times for removing any residual components.

**Table 1.** The composition of the copper hydroxide nitrate-based nanocomposite containing the Mo_6_ cluster as (1) ((C_4_H_9_)_4_N)_2_Mo_6_Cl_14_, (2) Cs_2_Mo_6_Cl_14_, (3) Cs_2_Mo_6_Br_14_, (4) Cs_2_Mo_6_I_14._

	Cu(NO_3_)_2_.3H_2_O (g)	(1) MC (g)	(2) MC (g)	(3) MC (g)	(4) MC (g)
(1)@CHN15	0.5	0.1			
(1)@CHN110	1.0	0.1			
(1)@CHN115	1.5	0.1			
(1)@CHN120	2.0	0.1			
(2)@CHN110	1.0		0.1		
(3)@CHN110	1.0			0.1	
(4)@CHN110	1.0				0.1

A referenced CHN was synthesized from the Cu(NO_3_)_2_∙3H_2_O compound using an ammonia solution as a catalytic agent. A homogeneous Cu(NO_3_)_2_∙3H_2_O solution in ethanol (30 g/L) was first treated at a temperature of 70°C for 30 min, then 0.2 ml of the NH_3_ solution was slowly dropped into the aqueous solution. The reaction to fabricate the CHN occurred for 2 h at 70°C. The powder was collected by centrifugation and washing several times with ethanol, then dried at 100°C for 24 h in air. All the powders were kept in a dry condition at room temperature for the next characterizations.

### 2.3. Sample characterization

X-ray powder diffraction (XRD) patterns were recorded at room temperature in the 2 theta range of 10°–60° using the Rigaku Smart Lab (Rigaku Corporation, Japan) 3 diffractometer (Cu Ka radiation) with a step size of 0.02° and a scan speed of 2° min^−1^. Le Bail fittings were performed using the FullProf program included in the WinPLOTR software. The zero-point shift, asymmetry parameters, and lattice parameters, and β angle were systematically refined, and the background contribution was manually estimated. The reflectance spectra of the powders and absorbance of the degraded dye solutions were measured by UV-Vis-NIR spectroscopy (V570, Jasco Corp., Japan) in the wavelength range of 220 to 2000 nm at the scan rate of 400 nm/min. The luminescent emitting spectra of the powders were measured by high-performance fluorescence spectroscopy (JASCO FP8500; Jasco Corporation, Japan) connected to a Xenon lamp at the scan rate of 500 nm/min. The surface morphology and elemental composition were analyzed by field emission scanning electron microscopy (FE-SEM, SU8000, Hitachi High-Technologies Corp., Japan) at 10 kV coupled with an energy-dispersive X-ray (EDX) analysis device. High-resolution observations of the powder were performed by HR-TEM (JEOL JEM 2100 F, JEOL Ltd., Japan) equipped with an EDX analysis device. The typical chemical vibration of the powder was verified by Fourier transform infrared spectroscopy (FTIR) (Thermo scientific Nicolet 4700, Thermo Fisher Scientific Inc., Japan) in the wavenumber range from 4000 to 400 cm^−1^ using a KBr pellet. The electron binding energy spectra within the MC and MC@CHN materials were measured by X-ray photoelectron spectroscopy (XPS) (PHI Quantera SXM (ULVAC-PHI), Inc., Japan) using Al Kα radiation at 20 kV and 5 mA and the taken-off the angle of 45°. All the binding energies were calibrated concerning the C 1s peak of the adventitious carbon at 285 eV. The magnetic property of the nanocomposite powders carried out in medical capsules was measured by using a superconducting quantum interference device (SQUID) magnetometer (Quantum Design (MPMS-XL), USA). The temperature in the range from 2 to 300 K was used to determine the dependence on the susceptibility in a magnetic field of 5kOe.

### 2.4. Catalytic degradation procedure

The catalytic activities of the MCs, CHN110 and MC@CHN110 compounds were evaluated by the oxidation degradation of methylene blue (MB) in an aqueous solution. All the catalytic reactions were performed using a 0.03 g catalyst in 20 ml of the MB aqueous solution (20 mg/L) without and with a specified dose of the activating H_2_O_2_ oxidizer (5, 10, and 15 mM). The weight of the MC catalyst (0.003 g) used as a reference corresponded to 10 wt% of the MC@CHN110. All the reactions were performed under magnetic stirring for 2 hours at room temperature in the dark. Afterward, 5.0 mL of the degraded dye solution was filtered by a hydrophobic plastic membrane (0.22 μm). The filtered MB was determined at the absorption peak of 664 nm, which is characteristic of MB in the UV-Vis absorption spectrum at the given time intervals of 30 min. The catalyst reusability was determined for the catalysts with an excellent removal efficiency through 4 cycles. The color removal efficiency (η, %) of MB was calculated using Eq. 1:

(1) $$\eta \left(\%\right)=\frac{\left(C_{0}-C_{t}\right)}{C_{0}}\times 100$$

where C_0_ is the initial concentration of MB and C_t_ is the concentration of MB after t min.

## 3. Results and discussions

### 3.1. The characterization of the MC@CHN powder

**Figure 2.**
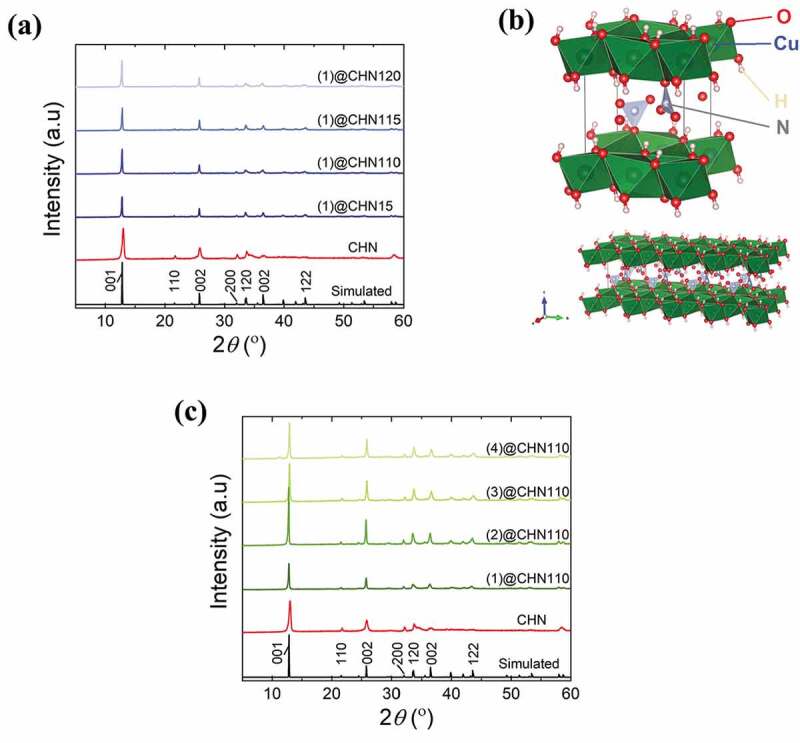
Powder XRD patterns of the CHN and their nanocomposites at a) different precursor compositions, b) The schematic illustration of single-layered copper hydroxynitrate (Cu_2_(OH)_3_NO_3_)), and c) powder XRD patterns of different nanocomposites using the A_2_[{Mo_6_X^i^_8_}X^a^_6_] starting MC precursor

A new preparation of the copper hydroxynitrate compound in the presence of the Mo_6_ cluster was investigated. Powder X-ray diffractograms of the MC@CHN nanocomposites with various MC and CHN weight ratios are displayed in Figure 2(a). The major reflection of the CHN structure (space group n°4, P121, *a* ≈ 5.605 Å, *b* ≈ 6.087 Å, *c* ≈ 6.929 Å, *β* ≈ 94.48°) can be observed. A Le Bail refinement of the whole samples has been performed and reasonably reliable factors have been obtained (Table 2). It confirmed that the MC@CHN nanocomposites mainly contain the monoclinic CHN crystal phase (Figure 2b). An example of refinement is displayed in Figure SI1 in order to illustrate the good correspondence between the experimental and simulated XRD pattern. These results are in agreement with the monoclinic CHN crystal phase from previous studies [30,31]. The crystallinity of the A_2_[{Mo_6_X^i^_8_}X^a^_6_] cluster almost disappears in the XRD diagram of the nanocomposite that suggests an amorphous MC phase mixed with the CHN crystals. The increase in the Cu precursor: MC ratio did not affect the crystallinity of CHN. Figure 2(c) shows the investigation to understand the influence of the chemical composition of the A_2_[{Mo_6_X^i^_8_}X^a^_6_] precursor (i.e. X and A) on the crystallinity of CHN. There is no significant difference in the CHN-assigned crystal peak whatever the A_2_[{Mo_6_X^i^_8_}X^a^_6_] starting precursor chemical composition. The Mo_6_ metallic core will be an important factor that determines the crystalline form of CHN without the effect of the inner and apical ligands.

**Table 2.** Lattices parameters and reliability factors obtained from the Le Bail refinement of XRD patterns of the MC@CHN110 nanocomposites

Cu_2_(OH)_3_(NO_3_); *P 1 21 1*; λ_Cu_ = 1.54056 Å; 300 K								
	*a* (Å)	*b* (Å)	*c* (Å)	*β* (°)	*V* (A^3^)	Chi^2^	R_p_	R_wp_
CHN	5.649(1)	6.126(10	6.980(1)	93.58(2)	241.1(1)	2.47	5.19	6.85
(1)@CHN110	5.586(1)	6.097(1)	6.863(2)	94.47(1)	233.1(1)	2.76	7.06	9.69
(2)@CHN110	5.603(1)	6.086(1)	6.924(2)	94.31(1)	235.4(1)	2.53	5.15	6.74
(3)@CHN110	5.609(1)	6.071(1)	6.934(2)	94.31(1)	235.4(1)	3.08	5.76	7.52
(4)@CHN110	5.602(1)	6.066(1)	6.930(2)	94.34(1)	234.9(1)	3.31	6.02	8.35

For a better understanding of the performance of CHN, the FT-IR spectra of the MC@CHN powders with different MC: CHN ratios were recorded (Figure SI2). As seen in the IR spectrum of **(1)**@CHN15 without washing, the vibrational peaks at 883, 784 and 676 cm^−1^ can be assigned to the hydrogen bonding frequencies related to the Cu-O-H bonding. In addition, the vibrational peaks contributed by the OH group also appear at 1663, 3443, and 3535 cm^−1^ in agreement with a previous publication. The IR peaks specific to the common NO_3_^−^ group are 813 (υ2), 1048 (υ1), and 1384 cm^−1^, while the peaks at 1334 and 1422 cm^−1^ can originate from the symmetric and asymmetric stretching mode of the NO_3_^−^ group occupied between the copper layer hydroxide. All the vibrational bands assigned to Cu_2_(OH)_3_NO_3_ are in agreement with previous reports [28,30,31]. The IR peaks that could be assigned to the Mo_6_ metallic core in the nanocomposite could not be assigned. However, the existence of the n-C_4_H_9_ counter cation in the **(1)**@CHN15 without washing was confirmed by the peaks at the frequencies of i) 2962, 2933 and 2877 cm ^−1^ contributed by the stretching mode of C-H; ii) 1378 and 1466 cm^−1^ contributed by the bending mode of C-H, and iii) 750 and 1163 cm^−1^ contributed by the bending mode of C-C. These characteristic peaks disappear after the **(1)**@CHN15 nanocomposite was purified. The IR spectra showed no difference in the chemical bonding caused by the various compositions of the nanocomposite. This result proved that the counterion of the A_2_[{Mo_6_X^i^_8_}X^a^_6_] cluster unit was separated from the [{Mo_6_X^i^_8_}X^a^_6_] metallic core during the mixing with CHN.

**Figure 3.**
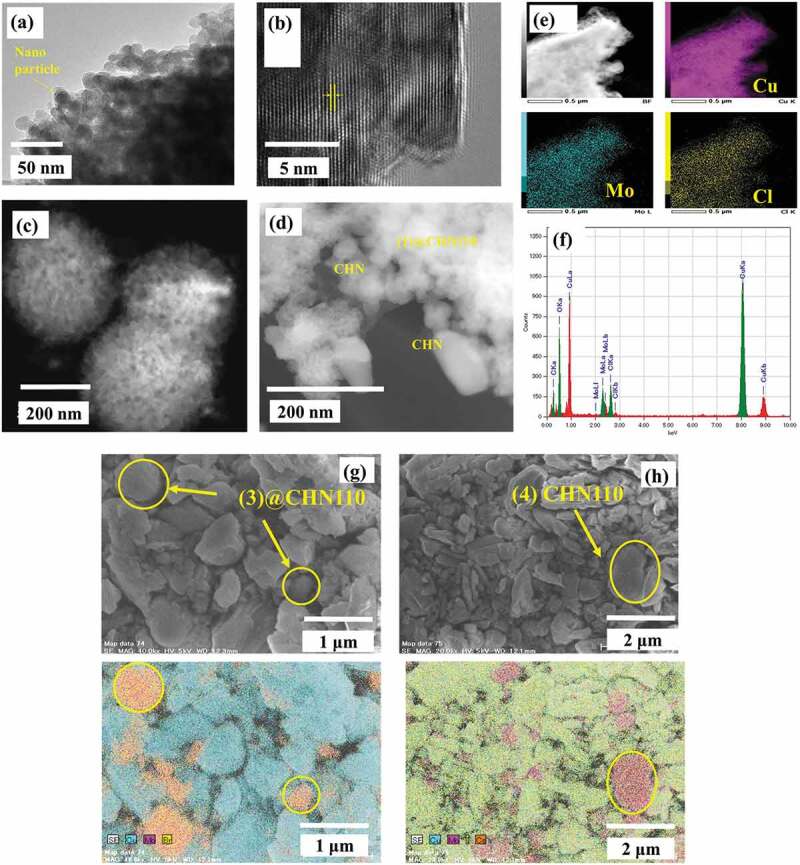
(a) TEM, b) HR-TEM, c) and d) STEM image, e) STEM-EDX mapping, and f) element spectrum of **(1)**@CHN110 powder. FE-SEM and FE-SEM-EDX mapping images of g) **(3)**@CHN110 and h) **(4)**@CHN110

The Cu_2_(OH)_3_NO_3_ nanocrystalline was recognized in the SEM image of (1)@CHN110 (Figure 3a) presenting the visible pattern as seen in the HR-TEM image (Figure 3b). This is in agreement with the X-ray diffractograms. After the first step of the reaction at RT for 24 h, the nanocomposite is in the form of a porous structure and a flower of nanometric size (Figure 3c). However, the efficiency to synthesize CHN at room temperature is poor, and difficult to collect the crystal product. For this reason, the thermal treatment at 80°C was applied for the slurry. The crystalline and amorphous phase mixed nanocomposite was recognized in Figure 3(d) with the CHN crystal blended with the amorphous M_6_ clusters. The element composition spectrum of the CHN and Mo_6_ cluster was confirmed again through the use of the STEM-EDX mapping, following the intercalation of the Cu_2_(OH)_3_NO_3_ and Mo_6_ cluster phases (Figure 3e and f). Similarly, the use of the (3) and (4) MC precursors to fabricate the nanocomposite also result in the amorphous and crystalline mixed-phases (Figure 3g and h).

**Figure 4.**
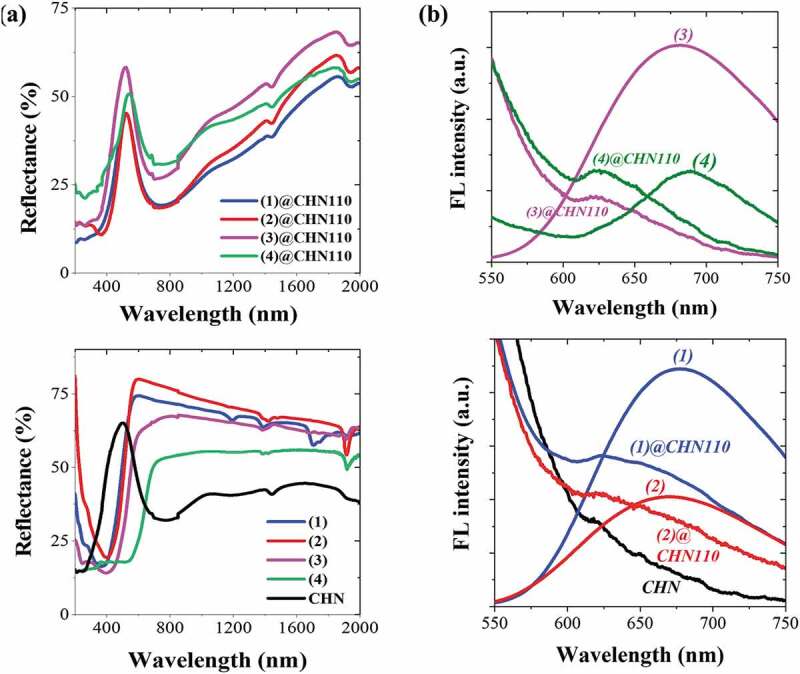
(a) The reflectance and (b) fluorescence spectra excited at 375 nm for the MC, CHN, and their nanocomposites

Aiming to confirm the existence of the integrity of the Mo_6_ octahedral structure after the thermal and chemical treatments, the optical absorption and emission spectra were studied. In the first investigation, the dependence of the optical property on the composition ratio between the MC and Cu precursor was studied (Figure SI3). The optical spectrum of CHN shows a strong absorption in the UV range below 300 nm, while the characteristic absorption of MC is observed below 400 nm. CHN also presents the relative absorption in the near-infrared range (NIR) from 600 to 900 nm (Figure SI3a). The spectrum of MC@CHN110 is composed of the optical absorption of both the MC and CHN components at the various weight ratios. However, the luminescent emission efficiency in the NIR range of the (1)@CHN decreases when the concentration of the Cu precursor increases (Figure SI3b). Considering this result, a large CHN crystal could increase the light absorption in the NIR range as seen in Figure SI3a that would reduce the light emission from **(1)** MC. The effect of the ligand of A_2_[{Mo_6_X^i^_8_}X^a^_6_] on the properties of CHN was also figured out. As is known, the optical properties of MC depend on the nature of the ligand which could be exchanged during the chemical and thermal treatments. Most of the MC@CHN110 show the UV-light absorption of the specified Mo_6_ clusters in the range lower than 400 nm and the NIR-light absorption of CHN at the peak of 735 nm. There is a shift to a lower wavelength when compared to the referenced CHN showing the peak at 750 nm (Figure 4a). Only (4) MC showed a big difference with a shifting of 200 nm to lower visible wavelengths when it was incorporated with CHN. This optical property modification noticed the strong change in the apical iodide ligand on (4) MC. It was also observed that the red color of the iodine molybdenum cluster almost changed to the emerald-green color in (4)@CHN110. The analyses of the fluorescence spectra will confirm the clear modification of the apical ligand of MC. CHN showed no emission in the NIR range while all the MC powders presented the obvious emission in the NIR range with the peaks at 677, 670, 679 and 688 nm assigned to the **(1), (2), (3)** and **(4)** MCs (Figure 4b), respectively [17]. However, the shape of the spectra that had changed consisted of an asymmetric structured broadband centered roughly at 622 nm for all the MC@CHN110 powders. This is explained by a significant exchange of the apical ligand of MC during the synthesizing steps. As is well known, the intensity of the photoluminescence of the Mo_6_ cluster decreased when the apical halogen ligands are partially replaced by ethanol, H_2_O molecules, or hydroxyl anions. Based on quantum chemical studies, Costuas et al. reported that the NIR photoluminescence originated from the important geometrical relaxations of the [{Mo_6_X^i^_8_}X^a^_6_]^2-^ based system occurring at the triplet state, depending on the outstretching of the Mo_6_ octahedron and the elongation of one Mo–Mo bond [9]. In addition, the external environment (counter-ions, crystal packing) of the cluster has a noticeable impact on its relaxation processes [9]. This is in agreement with the explanation that the separation of the counterion and the exchange of apical ligands by a water molecule causes modification of the NIR photoluminescence peak of the [{Mo_6_X^i^_8_}X^a^_6_]^2-^ cluster unit (X = Br, Cl, I).

Table 3 presents the bandgaps of the CHN, MCs, and MC@CHN110 powders calculated from the absorbance spectrum with the help of the Tauc plot (Figure SI4). Most MCs show a similar bandgap at about 2.2 eV (λ = 563 nm) following high activity in the visible light range. Only (4) MC showed a big difference in the energy bandgap (~ 0.4 eV) with others after introduced with CHN. This result agrees with the proper exchange of the iodide ligand during thermal treatment resulting in the modification of the photo bandgap.

**Table 3.** The energy bandgap (E_g_) of A_2_[{Mo_6_X^i^_8_}X^a^_6_] (A = Cs, or alkylammonium cations; X = Cl, Br, or I) determined by optical absorbance measurement

X	E_g_ (eV)	
	X	X@CHN110
CHN	2.55	-
(1)	2.19	2.16
(2)	2.15	2.15
(3)	2.15	2.00
(4)	1.62	2.00

The element component ratio of the nanocomposite was determined by using an HR-SEM coupled EDX device with a penetrated depth of about 1 μm (Figure SI5). The Mo/ligand ratios of the different Mo_6_ clusters (the theoretical value of 6/14) were confirmed in Figure SI5 and Table SI1. The Mo/ligand ratios determined for Mo/Cl**(1)**, Mo/Cl**(2)**, Mo/Br**(3)**, and Mo/I**(4)** were 6/13.5 ± 0.5, 6/13.3 ± 0.7, 6/11.4 ± 0.4, and 6/7.93 ± 0.5, respectively. The Mo/ligand ratio was calculated from the atom percentage of the Mo and ligand atoms indicated in the EDX spectrum. The error values of the ligand atom were calculated from 3 measurements at different positions on the surface of the samples. The apical Br and I ligands seem not to be stable in comparison to the Cl ligand. The Mo/Cl ratio is almost similar to the theoretical value (6/14) suggesting the stability of the Cl ligand of the [Mo_6_Cl_14_]^2-^ precursor during treatment of solvent and thermal conditions. These data are in agreement with the visible modification of the optical absorption and emission properties followed by the exchange of the apical ligand [2,9].

**Figure 5.**
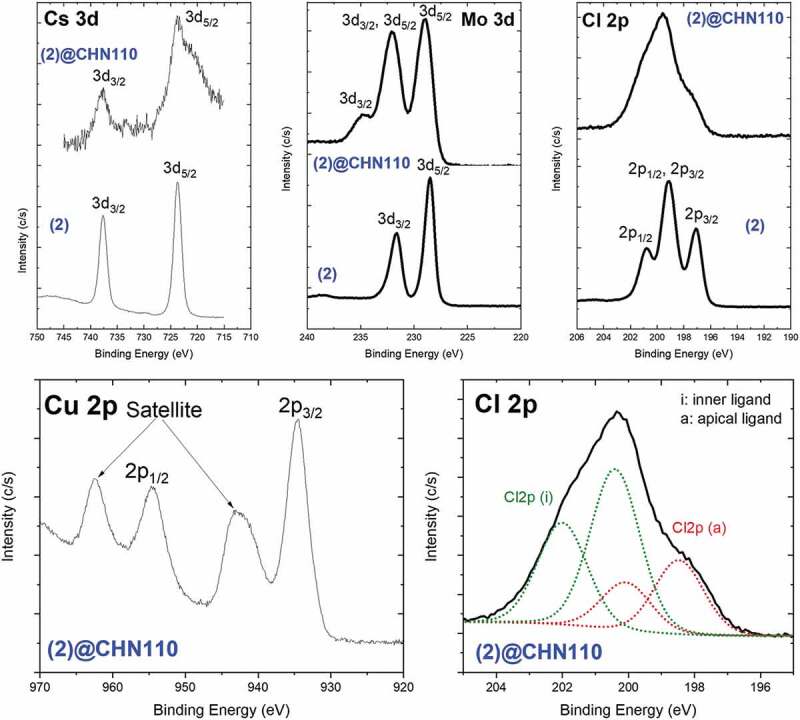
XPS binding energy spectrum of Cu 2p, Cs 3d, Mo 3d, and Cl 2p regions of the **(2)** and **(2)**@CHN110 nanocomposite. Deconvolution spectra of Cl 2p region of the **(2)**@CHN110 nanocomposite

For a deeper discussion about the intrinsic interaction between the Mo_6_ cluster and CHN as well as the confirmation of the CHN structure, X-ray photoelectron spectroscopy was performed for the Cs_2_[{Mo_6_Cl^i^_8_}Cl^a^_6_] **(2)** and **(2)**@CHN110 powders. All the binding energies were calibrated concerning the C1s peak of the adventitious carbon at 285 eV. The chemical confirmation of CHN was first determined based on the results of the binding energy and element compositions from the XPS survey scan spectrum of the (2)@CHN110 powder (Figure SI6). The Cu 2p binding energy peak at 935.30 (2p_1/2_) and 954.5 (2p_3/2_) was assigned to the Cu-O bonding of the CuOH group (Figure 5 and Table SI2). In addition, the measured Cu/N atomic ratio of about 2/1 seen in Table 4, the N1s binding energy peak at 407.2 eV assigned to NO_3_^−^, and the O1s binding energy peak at 531.4 eV assigned to OH seen in Tab. SI2 proves the existence of the Cu_2_(OH)_3_NO_3_ structure.

**Table 4.** The element compositions of the **(2) MC** and **(2)**@CHN110 powders were obtained from the XPS survey scan spectrum (Fig. SI6)

Sample	C 1s	N 1s	O 1s	Cl 2p	Cu 2p	Mo 3d	Cs 3d
	% atomic						
**(2) MC**	28.5	-	5.2	42.0	-	17.7	6.6
**(2)**@CHN110	23.8	5.5	47.7	6.8	11.3	4.8	0.1

As reported in a previous study of the Mo_6_@layer double hydroxide (Zn_2_Al), the appearance of the Mo-O-Zn or Mo-O-Al bonding between the Mo atom from the Mo_6_ cluster and O atom from the hydrotalcite layers of the LDH was confirmed by one new peak at 234 eV (3d_3/2_) indicating Mo-O bonding [11]. A similar result indicating the Mo-O bonding in this study was determined at 232.8 eV (Mo 3d_5/2_) and 235.6 eV (Mo 3d_3/2_). On the other hand, the Mo_6_ cluster could be partially oxidized during the synthesis of CHN to form the MoO_3_ compound. The Mo-O bonding is suggested to belong to the MoO_3_ compound or Mo-O-Cu new linking that is transformed from the Mo-Cl bonding (229.7 and 232.8 eV) of the Mo_6_ cluster (Figure 5 and Table SI2). Following the result of the total element compositions (Figure 5 and Table 4), the Cs atom is almost separated from the product during the washing procedure that was determined by a peak at 724.5 eV. The analysis of the inner and apical ligand is also important to confirm the stability of the Mo octahedron structure. The deconvolution spectrum of the Cl 2p region of the Mo_6_ cluster and in the nanocomposite similarly shows four peaks at 198.4, 200.0, 200.4 and 202.0 eV that indicate Cl^i^2p_3/2_, Cl^i^2p_1/2_, Cl^a^2p_3/2,_ and Cl^a^2p_1/2_, respectively (Figure 5 and Table SI3). The Table 4 results show that the total atomic ratio between the Mo and Cl atoms in the nanocomposite is about 6 and 8.5 while it is 6 and 14 for **(2) MC**. The total Cl ligand in the nanocomposite was lost during preparation due to the exchange of the apical ligand by the H_2_O or hydroxyl molecules or the formation of MoO_3_. In addition, the ratio of Mo, inner Cl, and apical Cl on the retained octahedral Mo_6_ cluster was measured at 6, 8 and 4 (Mo: Cl = 6:12) that was calculated from curve fitting of Mo 3d and Cl 2p (Figure 5) as illustrated in Table SI3. These results suggest a reduction of the two original apical Cl elements on the Mo_6_ cluster unit and neutral H_2_O or negative OH molecules can replace them to form a new cluster with the formula of [Mo_6_Cl^i^_8_Cl^a^_4_(OH)^a^_y_(H_2_O)^a^_x_]^x−2^ (x + y ≤ 2). In summary, based on the measured element concentration of copper and molybdenum in the nanocomposite, the expected chemical formula of the Mo_6_@CHN nanocomposite is predicted to be [{Mo_6_Cl^i^_8_}Cl^a^_4_(OH)^a^_y_(H_2_O)^a^_x_] ^x−2^@[Cu_2_(OH)_3_NO_3_]_7_.

**Figure 6.**
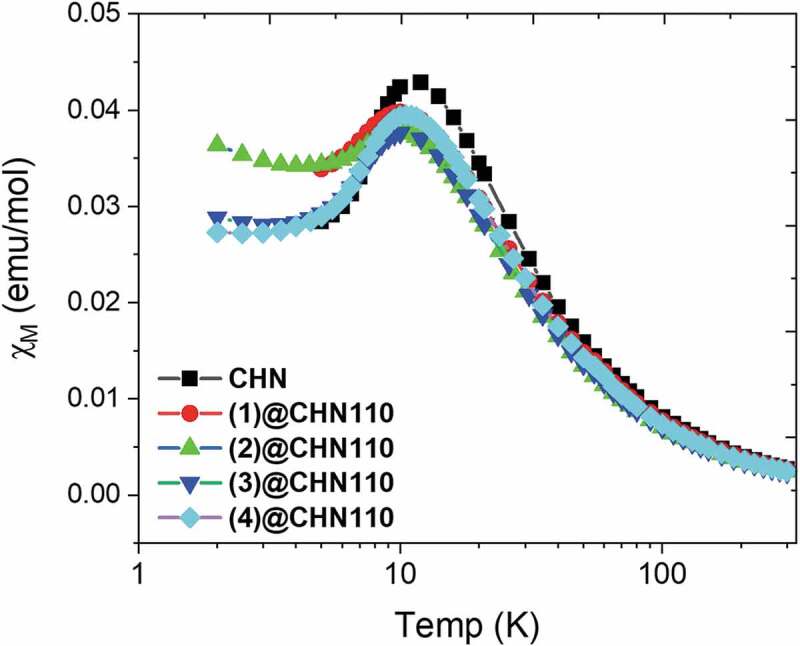
The temperature dependence of the CHN and MC@CHN110 nanocomposite on the molar magnetic susceptibilities (χ_M_) at 5kOe

Figure 6 presents the temperature dependence on the magnetic susceptibilities of the CHN and M_6_@CHN110 nanocomposite in the magnetic field of 5kOe. All the curves show the coincident shape at all temperatures without any significant difference between the CHN and Mo_6_@CHN110 powders. However, the T_max_ position at 11.6 K is assigned to CHN in agreement with previous studies [37,38]. This peak slightly shifts to a lower temperature at 10.3 K when the Mo_6_ cluster is added. In addition, the maximum value of the magnetic susceptibility χ_M_ of CHN decreases from 0.043 emu/mol to 0.039 emu/mol due to the effect of the Mo_6_ cluster. Depending on the Currie constant calculated from the temperature dependence on the magnetic susceptibilities, the spin number (S) of about 0.5, which was calculated in Table SI4, confirms the CHN and Mo_6_@CHN110 compounds containing only Cu^2+^ ion species. The redox reaction between the Mo_6_ cluster and copper (II) does not happen. In addition, the effective magnetic moment (P_eff_) of CHN and (1)@CHN is approximately about 2.5 while the other ones are slightly lower than the expected value (Tab. SI4). The magnetic susceptibility illustrates how much a material will become magnetized in an applied magnetic field with χ_M_ > 0 indicating the paramagnetic characteristic of CHN that is also obtained for the MC@CHN110 powders. The Mo-O-Cu possible covalent bonding will limit the magnetic moments of the electrons of the copper atom resulting in the reduction of the magnetic susceptibility. The reason for this interesting phenomenon should be determined in the future.

### 3.2. Study of the catalytic properties of the nanocomposite

**Figure 7.**
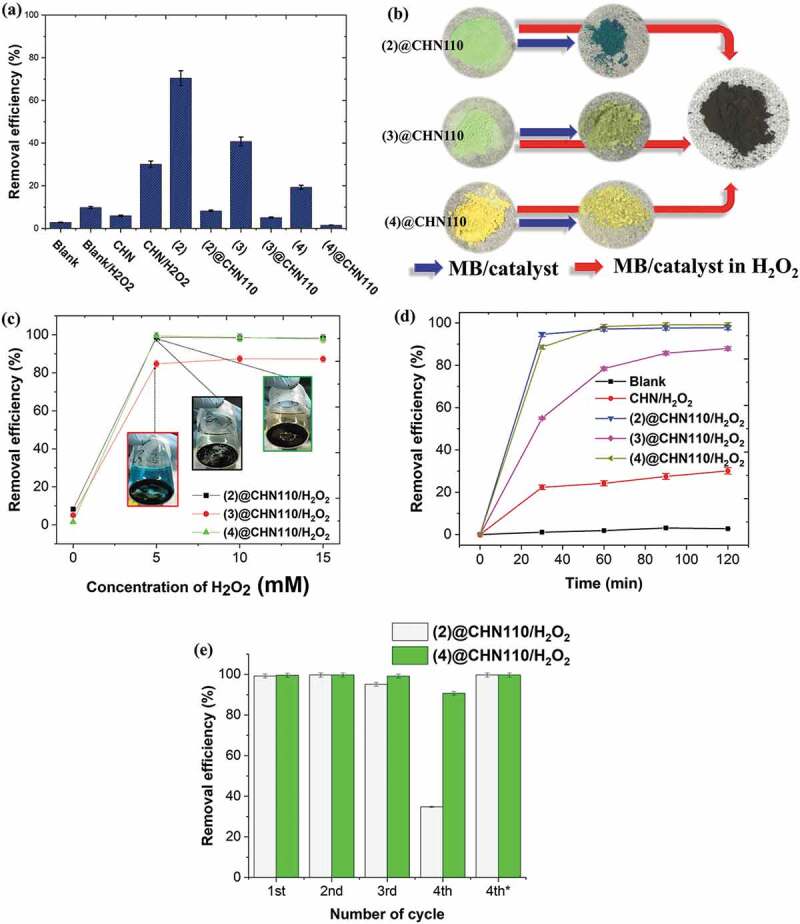
(a) The MB-degradable efficiency caused by the Mo_6_ cluster, and MC@CHN110 catalysts, b) the visual color of the catalyst before and after reacting with the MB dye c) the effect of the H_2_O_2_ concentrations on the MB-degradable efficiency by using MC@CHN110, d) the decolorization rate at the H_2_O_2_ concentration of 10 mM, and e) catalyst reuse using the MC@CHN110/H_2_O_2_ catalytic systems with the 4^th^* symbol denoting the filtered MB solution remeasured from the 4^th^ cycling solution after 3 days. All the reactions were performed for 2 h in the dark

The decolorization performances of the aqueous methylene blue (MB) solution, as the model of an organic pollutant, using the MC, CHN, and MC@CHN110 catalysts were evaluated for 2 h at RT in the dark (Figure 7a). The colour removal efficiency was calculated from Eq. 1. It was noted that the MB decolorization by using only H_2_O_2_ or CHN is not efficient with a value recorded lower than 10%. However, the catalytic property of CHN is significantly accelerated by using an H_2_O_2_ oxidizer with the MB removal efficiency reaching about 30%. The investigation of the catalytic efficiency was performed on different cluster precursors, A_2_[{Mo_6_X^i^_8_}X^a^_6_], with the different ligands (Figure 7a). The Cs_2_[{Mo_6_Cl^i^_8_}Cl^a^_6_] **(2**), Cs_2_[{Mo_6_Br^i^_8_}Br^a^_6_] **(3)** and Cs_2_[{Mo_6_I^i^_8_}I^a^_6_] **(4)** result in the MB removal efficiency corresponding to 70, 40, and 20%, respectively, that shows a better dye removing possibility than CHN. A similar tendency of dye removal efficiency caused by the MCs also occurred in combination with CHN. The photos of the MB solutions after reacting with a specialized catalyst are presented in Figure SI7. In Figure 7(b), the blue-colored MB existing on the surface of (2)@CHN110 was recognized and it is significantly reduced for (3)@CHN110 and (4) @CHN110. It means that the MB-concentration reduction depends on both adsorption and catalytic processes in the case of the nanocomposite. As is known, the catalytic characteristic of the MCs was partially affected by the nature of the ligands, and it is reduced from the Cl, Br and I ligand that contributes to the possibility of generating a pair of holes and electrons on the cluster [9,17]. Even though the catalytic property of itself cluster is relative, the reuse of the MCs faces a big difficulty. For this reason, finding suitable supporting material like CHN to improve the reuse of the MCs is one of the main goals of this study. Interestingly, the MB color visually observed completely disappears when it reacts with the MC@CHNb catalyst activated by H_2_O_2_ resulting in a brown-colored powder, i.e. a product containing Cu (I) and Cu (II) elements. This result confirmed that the H_2_O_2_ activated nanocomposite completely reduced the blue-colored MB or the catalytic reaction plays a crucial role to degrade the dye without an adsorption mechanism.

An investigation of the activation of H_2_O_2_ on the MC@CHN110 compounds was then performed at different H_2_O_2_ concentrations as presented in Figure 7(c). The dye removal efficiency values caused by the (2)@CHN110 and (4)@CHN110 are impressive at more than 98% in comparison with another one even for the minimum H_2_O_2_ concentration (5 mM). These values are in agreement with the photos of the visually degraded MB color. The **(3)** @CHN110 **/H_2_O_2_** system presents the catalytic possibility with the saturated removal efficiency higher than 85% at the maximum H_2_O_2_ concentration (15 mM). It is interesting to obtain a high removal efficiency by using Cs_2_[{Mo_6_I^i^_8_}I^a^_6_]@CHN110 even though Cs_2_[{Mo_6_I^i^_8_}I^a^_6_] shows a weak catalytic activity in comparison to the other ones. At a high concentration of H_2_O_2_, the dye solution contains plenty of bubbles caused by the degradation reaction by the residual H_2_O_2_. It is concluded that the catalytic activity of the catalyst by the acceleration of H_2_O_2_ completely contributes to the degradation of the dye without adsorption.

The study of the MB removal efficiency as a function of the reacting time with an interval time of 30 min was carried out for the MC@CHN110/H_2_O_2_ (10 mM) catalyst systems as seen in Figure 7(d). The calculated results were based on the optical absorption spectra of the filtered-MB solution after reacting with the catalysts shown in Figure SI8. Interestingly, the MB degrading efficiency caused by the (2)@CHN110/H_2_O_2_ and (4)@CHN110/H_2_O_2_ catalysts was about 90% in the first 30 minutes, then reached almost 100% after 1 h. In the case of (3)@CHN110/H_2_O_2_, the MB degrading efficiency value reached 83% after the oxidizing reaction was performed for 2 h. The MC@CHN110/H_2_O_2_ catalyst group shows a remarkable catalytic efficiency in comparison to CHN activated by the H_2_O_2_ oxidizer which shows a value of 30% (Figure 7a).

The activity and stability of the MC@CHN110 nanocomposite were further investigated by four continually recycling runs. The catalyst reuse was carried out using the MC@CHN110 activated by H_2_O_2_ (Figure 7e). The Cs_2_[{Mo_6_Cl^i^_8_}Cl^a^_6_] **(2)** and Cs_2_[{Mo_6_I^i^_8_}I^a^_6_] **(4)** functionalized CHN110/H_2_O_2_ systems were selected to evaluate the reuse due to their high dye removal efficiency that was based on Figure 7(c and d). The MB degradation efficiency by using the selected catalyst reached a similar remarkable value of 98% after three cycles. While the Cs_2_[{Mo_6_I^i^_8_}I^a^_6_] **(4)** functionalized CHN110/H_2_O_2_ systems still retained a high removal efficiency at 90% at the fourth recycling, the Cs_2_[{Mo_6_Cl^i^_8_}Cl^a^_6_] **(2)** functionalized CHN110/H_2_O_2_ systems only had about a 32% efficiency. When the reaction was kept running in the dark for 3 days, the MB degradation had a maximum color removal efficiency like the first cycle. These results strongly support the fact that the MC@CHN110 activated by H_2_O_2_ can be a powerful catalyst to degrade the MB dye with a high reuse efficiency.

**Figure 8.**
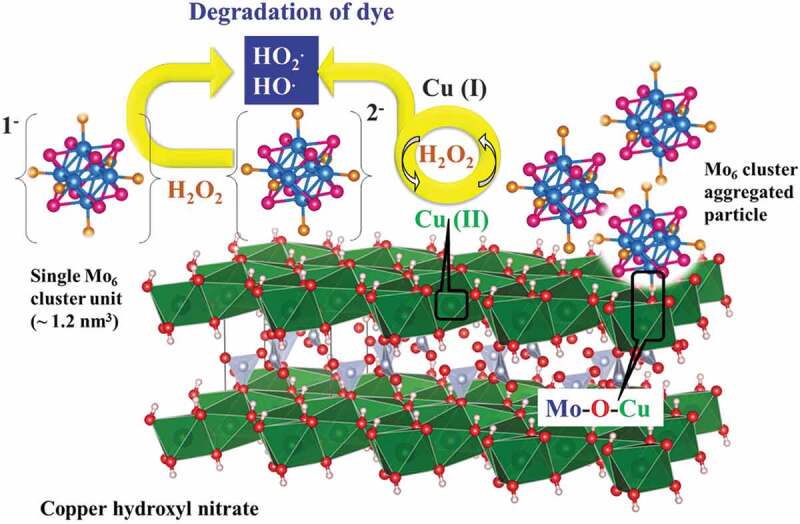
Schematic representation of the interaction between the components and the heterogeneous activation mechanism of the Mo_6_@copper hydroxyl nitrate initated by the H_2_O_2_ oxidation agents

Both the Mo_6_ cluster and copper hydroxynitrate produced an individually weak catalytic ability for the MB degradation but they show a specific role in the nanocomposite. Following the proposed model, the Mo_6_ cluster containing 24 electrons in the valence band is easy to be oxidized by hydrogen peroxide (H_2_O_2_) to form the hydroxyl radical (HO^·^) as illustrated in Eq. 2. The [Mo_6_X_14_]^−1^ cluster anion is also known as a powerful oxidant that contributes to decomposing the dye [39]. The hydrogen peroxide will reduce copper (II) to copper (I) hydroxide through the formation of copper (II) hydroperoxide as an intermediate agent following Eqs. 3 and 4 [40]. The appearance of copper (I) was noticed in the immediate color change from green to brown after the hydrogen peroxide was added to the reaction solution. Following Eq. 5, the copper (I) hydroxide can then decompose the H_2_O_2_ to form the powerful oxidation agent as the hydroxyl free radical and simultaneously return to the copper (II) hydroxide [40,41]. Finally, the hydroxyl radical and [Mo_6_X_14_]^−1^ oxidant will decompose MB to generate the degradation products (Eq. 6). The reversion between Cu (I) and Cu (II) hydroxide possibly happens with the acceleration of H_2_O_2_ that will be a remarkable point for the reuse for the catalytic application field. Eqs. 7 and 8 possibly occur to form the reduced Mo_6_ cluster. The representation of the structure and activity mechanism of Mo_6_@CHN for the degradation of MB is illustrated in Figure 8. In summary, H_2_O_2_ will activate the catalytic property of the Mo_6_ cluster and copper hydroxynitrate to produce the radical hydroxyl ion resulting in a high improvement of the dye removal reaction. A high activating efficiency of H_2_O_2_ for the catalytic property of the Mo_6_ cluster was also found in an MC/Zn-Al layered double hydroxide (LDH) nanocomposite as previously reported [11]. In comparison with the last combination of the Mo_6_ cluster and LDH, MC@CHN produces a faster catalytic efficiency and improvement of the reuse without the support of the adsorption phenomena. CHN, the Mo_6_ compound, and H_2_O_2_ have distinct roles to produce the proper catalytic property for this new nanocomposite. It is suggested that i) H_2_O_2_ will activate the catalytic property of the Mo_6_ cluster and CHN; ii) the Mo_6_ cluster plays an essential role to create the hydroxyl radical generated from H_2_O_2_, iii) CHN enhances the concentration of the hydroxyl radical generated from H_2_O_2_, and iv) the Mo_6_ cluster and CHN support each other to return to the original oxidizing state for the improvement of the catalytic reuse. The copper hydroxynitrate will be a promising supporting material to improve the collection of the Mo_6_ cluster after the catalytic reaction finishes.

(2) $$\begin{array}{rl} & H_{2}O_{2}+{\left(Mo_{6}cluster\;unit\right)}^{2-}\\ & \;\;\;\;\;\;\;\;\;\;\;\;\;\;\;\;\;\;\;\;\;\;\;\;\;\;\;\to {\left(Mo_{6}cluster\;unit\right)}^{-}+HO^{\cdot }+OH^{-} \end{array}$$

(3) $$H_{2}O_{2}+Cu^{2+}\to CuOOH^{+}+H^{+}$$

(4) $$CuOOH^{+}\to {O_{2}}^{-}+H^{+}+Cu^{1+}$$

(5) $$Cu^{1+}+H_{2}O_{2}\to HO^{\cdot }+OH^{-}+Cu^{2+}$$

(6) $${O_{2}}^{\cdot }or\;HO^{\cdot }+MB\to degradation\;products$$

(7) $${\left(Mo_{6}\;cluster\;unit\right)}^{-}+{O_{2}}^{\cdot }\to {\left(Mo_{6}\;cluster\right)}^{2-}+O_{2}$$

(8) $$\begin{array}{rl} & {\left(Mo_{6}\;cluster\;unit\right)}^{-}+Cu^{1+}\\ & \;\;\;\;\;\;\;\;\;\;\;\;\;\;\;\;\;\;\;\;\;\;\;\;\;\;\;\;\;\;\;\to {{\left(Mo_{6}\;cluster\;unit\right)}^{2}}^{-}+Cu^{2+} \end{array}$$

## 4. Conclusions

A nanocomposite MC@CHN composed by the Mo_6_ cluster units and copper hydroxynitrate was successfully synthesized by a colloidal chemical process combined with a thermal treatment step. Locally, the CHN crystallites are mixed by the nano-sized Mo_6_ cluster units. Chemical interactions between the MC and CHN were proved based on the binding energy spectrum. The investigation of the oxidation properties of the MC@CHN composites was demonstrated through the degradation of methylene blue. A high catalytic degradation rate after 30 minutes was reached by using the MC@CHN/H_2_O_2_ systems. The reuse of the system was demonstrated up to 4 reaction cycles with an excellent efficiency. A catalytic mechanism based on the Mo_6_ cluster and copper hydroxynitrate was reasonably proposed. On the one hand, it has excellent UV-NIR blocking properties and, on the other hand, it has the excellent ability to degrade organic pollutants that are potentially promoted inside buildings.

## Supplementary Material

Supplemental Material
