# Supplementary material for: Synthesis of novel hexamolybdenum cluster-functionalized copper hydroxide nanocomposites and its catalytic activity for organic molecule degradation
Source: Sci Technol Adv Mater. 2021 Sep 15;22(1):758–71. doi: 10.1080/14686996.2021.1961559 (PMC8463035; doi:10.1080/14686996.2021.1961559)
Supplement: Supplemental Material [file TSTA_A_1961559_SM9113.pdf]

SUPPLEMENTARY INFORMATION

Synthesis of novel hexamolybdenum cluster-functionalized copper hydroxide nanocomposites and its catalytic activity for organic molecule degradation

Thi Kim Ngan Nguyen[a,b], Cédric Bourgès[c], Takashi Naka[a], Fabien Grasset[a,b], Noée Dumait[d], Stéphane Cordier[d], Takao Mori[c], Naoki Ohashi[a,b], Tetsuo Uchikoshi[a,b]

[a]Research Center for Functional Materials, National Institute for Materials Science (NIMS), 1-2-1 Sengen, Tsukuba, Ibaraki 305-0047, Japan
[b]CNRS–Saint-Gobain–NIMS, IRL3629, Laboratory for Innovative Key Materials and Structures, National Institute for Materials Science, 1-1 Namiki, Tsukuba, Ibaraki 305-0044, Japan
[c]WPI International Center for Materials Nanoarchitechtonics (WPI-MANA), National Institute for Materials Science (NIMS), Namiki 1-1, Tsukuba, Japan
[d]Univ. Rennes-CNRS-Institut des Sciences Chimiques de Rennes, UMR6226, 35000 Rennes, France

Table SI1. The element ratio of Mo and ligand atoms by using the SEM-EDX mapping with the penetrated depth of about 1 μm.

Sample	Mo	Ligand
Theoretical	6	14
(1)@CHN110	6	13.5 ± 0.5
(2)@CHN110	6	13.3 ± 0.7
(3)@CHN110	6	11.4 ± 0.4
(4)@CHN110	6	7.93 ± 0.5

Table SI2. XPS binding energy (eV) of the $Cs_2Mo_6Cl_{14}$ (2) powder and the (2)@CHN110 nanocomposite.

Element		**$Cs_2Mo_6Cl^i_8Cl^a_6$ (2)** Binding energy (eV)	(2)@CHN110 Binding energy (eV)
Cs 3d	$3d_{5/2}$	724.9	724.5
	$3d_{3/2}$	738.8	738.8
Mo 3d	$3d_{5/2}$	229.7 (Mo-Cl)	229.7 (Mo-Cl); 232.8 (Mo-O)
	$3d_{3/2}$	232.8 (Mo-Cl)	232.8 (Mo-Cl, 235.6 (Mo-O)
Cl 2p	$2p_{3/2}$	198.25 (Cl^a); 200.39 (Cl^i)	198.47 (Cl^a); 200.40 (Cl^i)
	$2p_{1/2}$	199.85 (Cl^a); 201.99 (Cl^i)	200.07 (Cl^a); 202.00 (Cl^i)
Cu 2p	$2p_{3/2}$		935.30 (Cu-OH (Hydroxide))
	$2p_{1/2}$		954.51
N 1s			407.2 (NO_3^-)
C 1s		285.0; 286.4; 289.1	285.0; 286.3; 288.9
O 1s		532.6 (H_2O)	532.4 (H_2O); 531.4 (Cu-OH)

Table SI3. XPS binding energy (eV) of deconvolution spectra of the Mo 3d and Cl 2p regions of the $[Mo_6Cl^i_8Cl^a_6]^2$ anions of (2) cluster and (2)@CHN110 nanocomposite.

	Peak	eV	FWHM	%Gauss	%area	Peak
Cl2p<br>**(2)**	1	198.25	1.15	100	27.88	Cl2p3(a)
	2	199.85	1.15	100	14.08	Cl2p1(a)
	3	200.39	1.16	100	37.78	Cl2p3(i)
	4	201.99	1.16	100	20.26	Cl2p1(i)
Cl2p<br>(2)@CHN110	1	198.47	1.77	90	19.27	Cl2p3(a)
	2	200.07	1.77	90	12.60	Cl2p1(a)
	3	200.40	1.77	90	41.64	Cl2p3(i)
	4	202.00	1.77	90	26.49	Cl2p1(i)
Mo3d<br>(2)@CHN110	1	229.70	1.65	95	41.69	Mo-Cl(Mo cluster):<br>Mo 3d5
	2	232.80	2.05	79	46.19	Mo-Cl(Mo cluster):<br>Mo 3d3<br>MoO_3: Mo 3d5
	3	235.60	2.05	62	12.12	MoO_3: Mo 3d3

Table SI4. The Curie constant, spin number, and effective magnetic moment of the powders.

Powder	Curie constant	S	P_{eff} (effective magnetic moment)
CHN	0.831	0.54	2.579
(1)@CHN110	0.797	0.52	2.525
(2)@CHN110	0.739	0.49	2.432
(3)@CHN110	0.711	0.48	2.384
(4)@CHN110	0.740	0.49	2.434

* S = 1 (Cu^{3+}), S = ½ (Cu^{2+}), S = 0 (Cu^{1+})

FIGURES

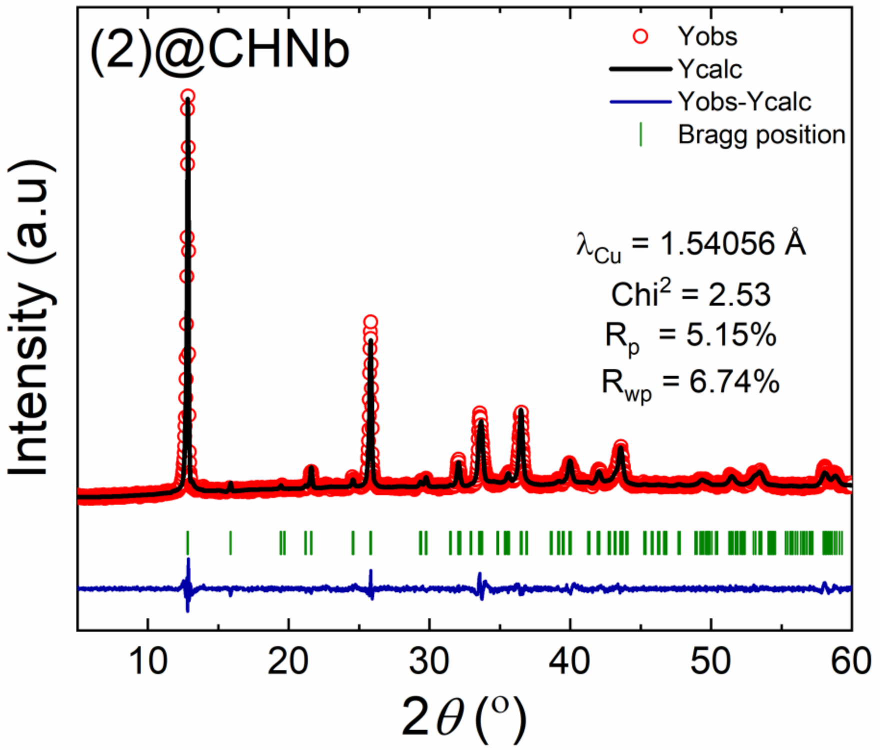

Figure SI1. Illustrative Le Bail refinement (Space group n°4, *P* 1 21 1, *a* = 5.603 Å, *b* = 6.086 Å, *c* = 6.924 Å, *β* = 94.31°) of the (2)@CHN110 sample.

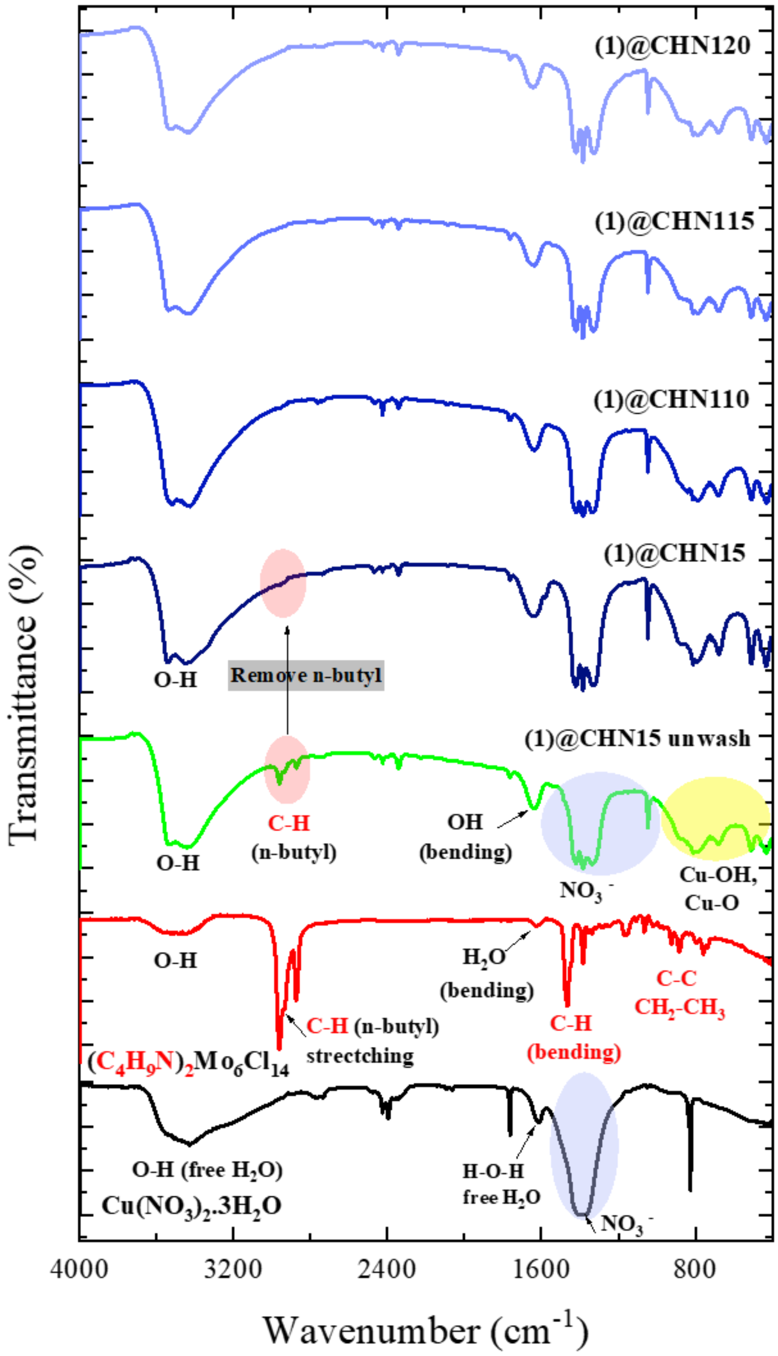

Figure SI2. FT-IR spectra of MC, CHN, and their nanocomposites at different compositions.

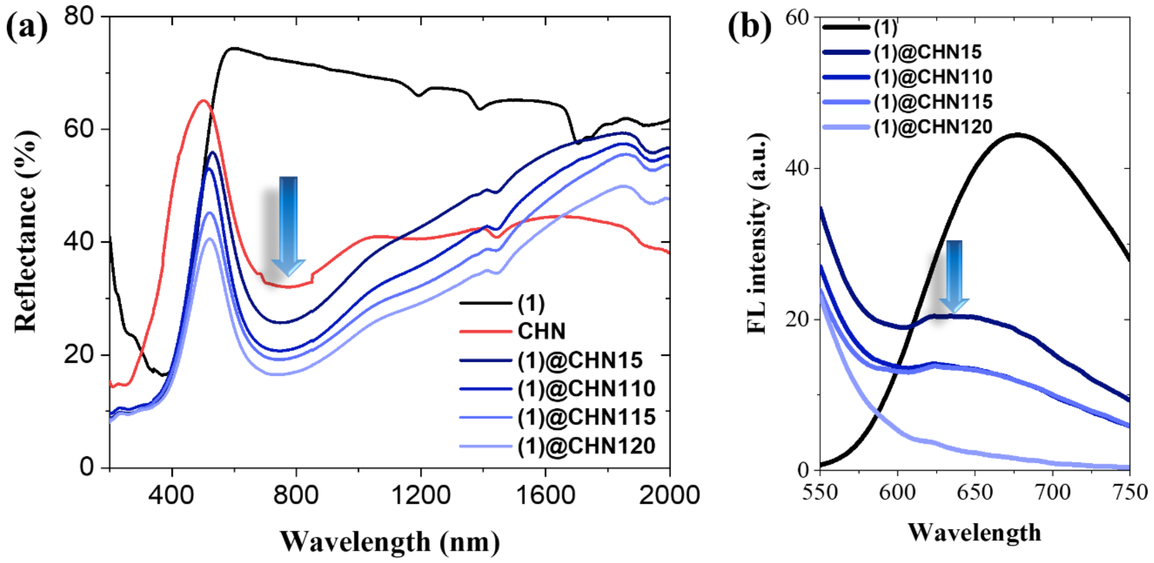

Figure SI3. a) The reflectance and b) fluorescence spectra of MC, CHN, and their nanocomposites at different compositions.

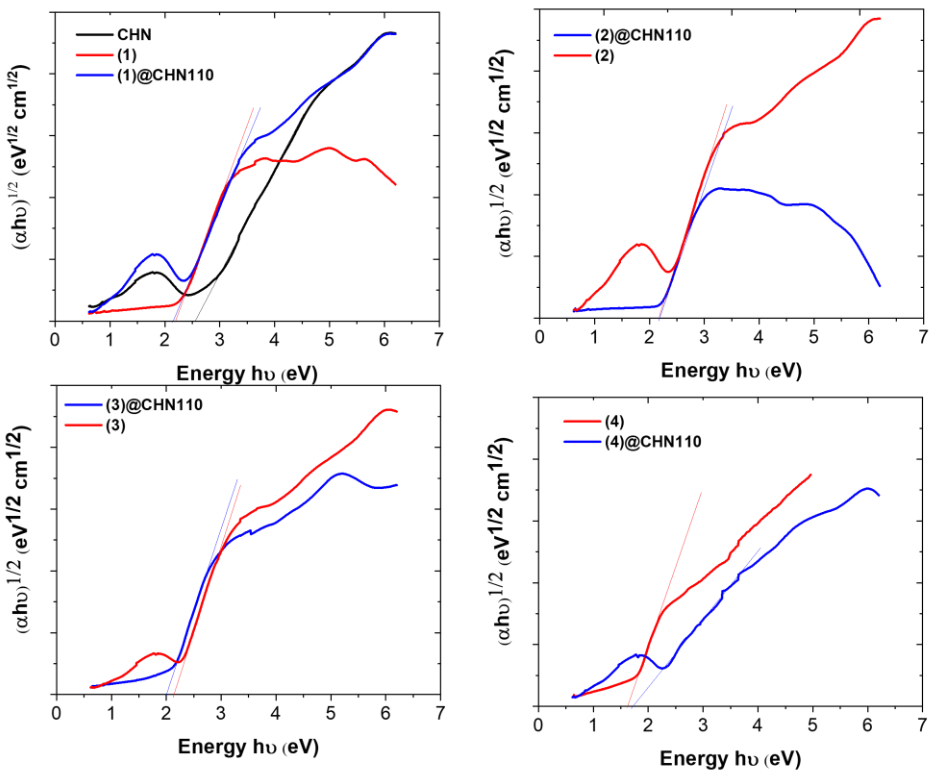

Figure SI4. Tauc plots for optical band gap determination of the MCs, CHN, and MC@CHN110 nanocomposites.

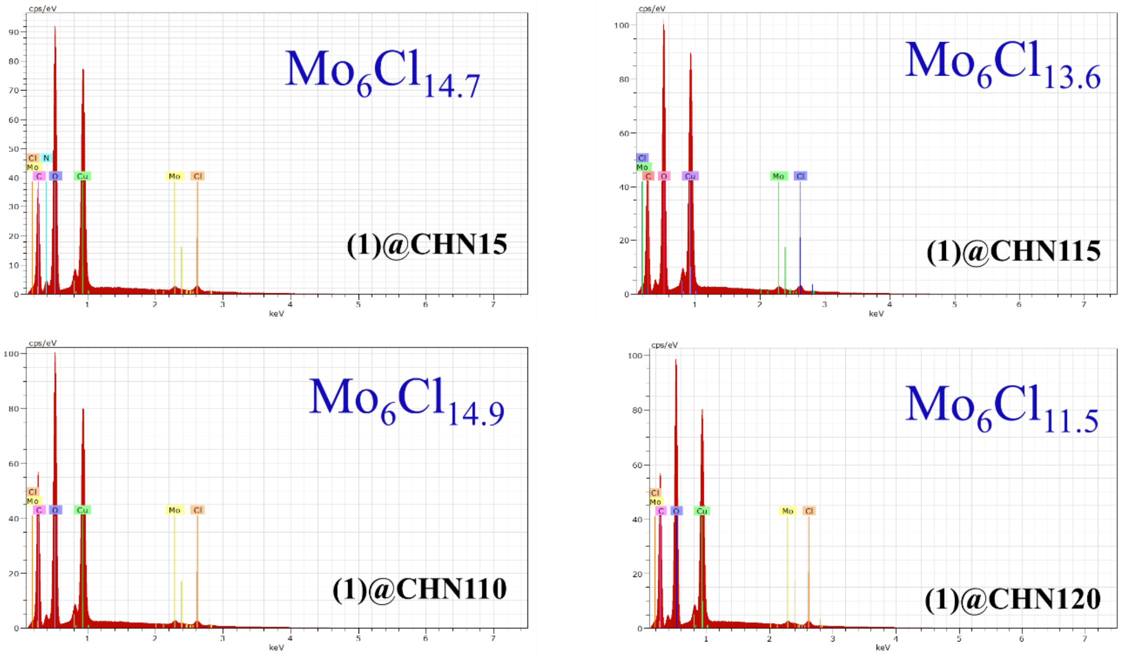

Figure SI5. a) Element composition spectra of MC@CHN nanocomposites at different reaction ratios by using HR-SEM coupled EDX device.

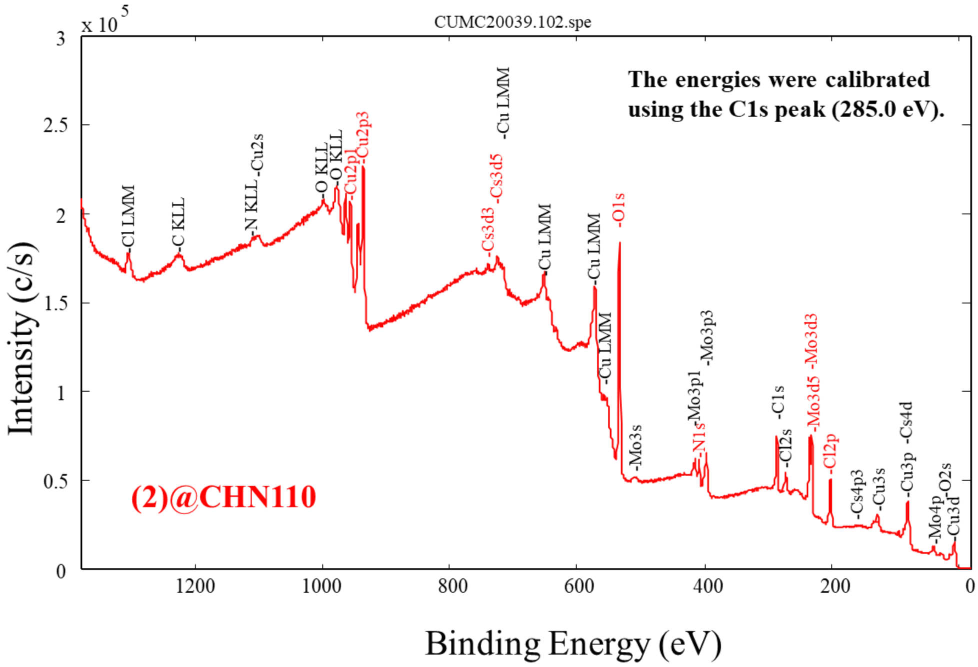

Figure SI6. XPS survey scan spectrum of the (2)@CHN110 powder.

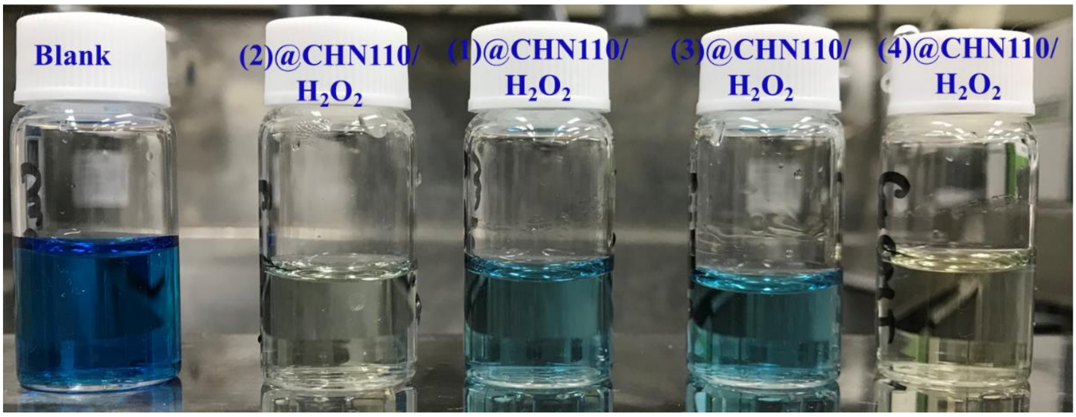

Figure SI7. The photos of the MB reduced solutions after reacting with the different catalysts.

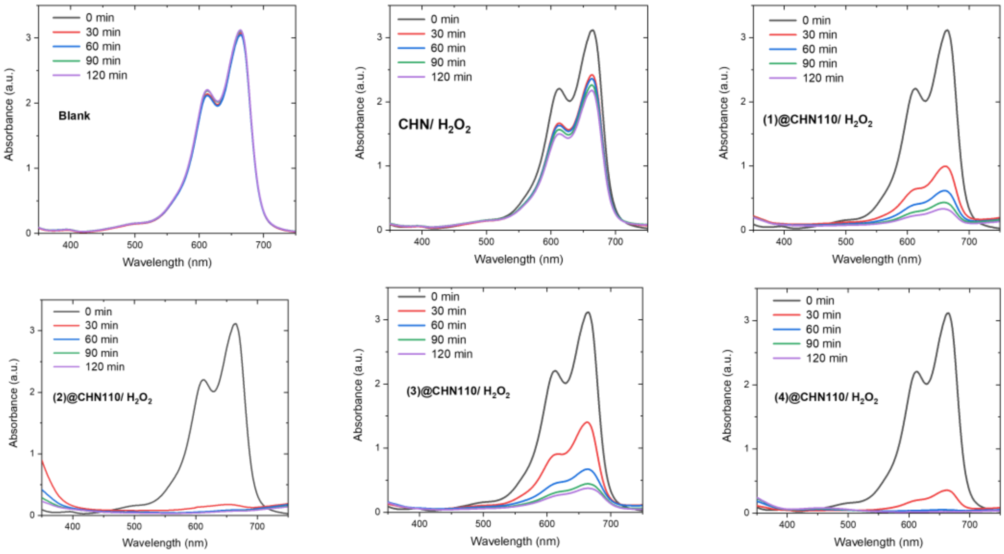

Figure SI8. The optical absorption spectra of the filtrated-MB solution using different catalysts.
